# Evaluation of a Remote Patient Monitoring Program During the COVID-19 Pandemic: Retrospective Case Study With a Mixed Methods Explanatory Sequential Design

**DOI:** 10.2196/55732

**Published:** 2024-07-09

**Authors:** Rose Gunn, Shelby L Watkins, Dave Boston, A Gabriela Rosales, Stefan Massimino, Suparna Navale, Stephanie L Fitzpatrick, John Dickerson, Rachel Gold, George Lee, Carmit K McMullen

**Affiliations:** 1 OCHIN, Inc Portland, OR United States; 2 Kaiser Permanente Center for Health Research Kaiser Permanente Portland, OR United States; 3 Asian Health Services Oakland, CA United States

**Keywords:** Patient-generated health data, telemedicine, telehealth, diabetes mellitus, hypertension, self-management, patient portal, implementation science, COVID-19: pandemic, community health center, chronic condition, remote patient monitoring

## Abstract

**Background:**

Community health center (CHC) patients experience a disproportionately high prevalence of chronic conditions and barriers to accessing technologies that might support the management of these conditions. One such technology includes tools used for remote patient monitoring (RPM), the use of which surged during the COVID-19 pandemic.

**Objective:**

The aim of this study was to assess how a CHC implemented an RPM program during the COVID-19 pandemic.

**Methods:**

This retrospective case study used a mixed methods explanatory sequential design to evaluate a CHC’s implementation of a suite of RPM tools during the COVID-19 pandemic. Analyses used electronic health record–extracted health outcomes data and semistructured interviews with the CHC’s staff and patients participating in the RPM program.

**Results:**

The CHC enrolled 147 patients in a hypertension RPM program. After 6 months of RPM use, mean systolic blood pressure (BP) was 13.4 mm Hg lower and mean diastolic BP 6.4 mm Hg lower, corresponding with an increase in hypertension control (BP<140/90 mm Hg) from 33.3% of patients to 81.5%. Considerable effort was dedicated to standing up the program, reinforced by organizational prioritization of chronic disease management, and by a clinician who championed program implementation. Noted barriers to implementation of the RPM program were limited initial training, lack of sustained support, and complexities related to the RPM device technology.

**Conclusions:**

While RPM technology holds promise for addressing chronic disease management, successful RPM program requires substantial investment in implementation support and technical assistance.

## Introduction

Chronic conditions including type 2 diabetes mellitus and hypertension (HTN) affect 6 in 10 adults in the United States and are leading contributors to disability and mortality [1,2]. Community health centers (CHC) serve low-income populations whose members experience a disproportionately high prevalence of these conditions [3]. CHC patients also face barriers to accessing and using promising new technologies that can improve chronic disease management, such as telehealth and home monitoring devices [4-6]. Varying terminology is used for such technologies. Here, “Remote Patient Monitoring” (RPM) describes patient-operated external biometric measurement devices with associated software and hardware to allow for patient-facing reporting and electronic health record (EHR) integration, used to record personal health data in one location that is received by a provider elsewhere [7].

RPM has been shown to benefit patients and their care teams by improving access to care, supporting clinical decision-making, and promoting chronic disease self-management [8-13]. Its use has also been associated with improved health outcomes, reduced emergency department visits and hospital admissions, and lower costs of care [14-16]. Despite these promising potential benefits, little has been reported on how best to implement RPM programs in primary care settings and what elements of RPM effectively engage patients and clinic staff [12]. This is particularly significant in health centers like CHCs, which serve a high volume of patients who are uninsured or Medicaid-insured for whom health disparities might be exacerbated by health centers’ delayed adoption of digital health technologies [17].

The Federal Communications Commission’s (FCC’s) COVID-19 Telehealth Program distributed $200 million under the CARES (Coronavirus Aid Relief and Economic Security) Act to help community-based health care organizations [18]. The program supported the provision of telehealth services and Wi-Fi–connected devices, including those used for RPM. This provided a unique opportunity to understand how CHCs implemented an RPM program during a period when in-person care was greatly restricted.

OCHIN, a nonprofit health technology organization hosting a central instance of the OCHIN Epic EHR system, received FCC COVID-19 Telehealth Program funds to distribute technology to support RPM to 24 health centers. The technology included Bluetooth-enabled glucometers, blood pressure (BP) cuffs, weight scales, pulse oximeters, and smartphones with 1 year of prepaid wireless data. OCHIN offered the 24 participating health centers support with distributing these devices through a help desk, device setup and troubleshooting videos, printed guides for patients and clinic staff, and facilitation of a monthly peer-to-peer learning collaborative meeting. Health centers selected which of the offered devices to distribute, determined which of their patients would receive them, and decided whether the devices should be given to patients permanently or temporarily and then redistributed. The RPM programs were implemented in different ways at the participating sites, one of which stood out as a success story. This manuscript describes implementation of the RPM program in this health center, including identified barriers and facilitators to RPM adoption, and patterns of improvement in participating patients’ BP.

## Methods

### Overview

We used a mixed methods explanatory sequential design to evaluate one CHC’s implementation of an RPM program made available through the FCC COVID-19 Telehealth Program [19]. When we gathered information about the FCC grant program, we learned from informal interviews and formal program evaluation data that the program did not generally result in successful RPM adoption. Using a positive deviance case study design [20,21], we identified one participating CHC that experienced unusual success in distributing and gathering data from RPM devices. These analyses quantitatively evaluate the program’s impact on BP and qualitatively explain that impact through interviews with CHC personnel and patients.

### Study Setting

The setting was a single CHC health system that implemented an RPM program in fall 2020. This urban CHC system provides wraparound services (eg, medical, dental, and behavioral health) to over 50,000 patients, most of whom speak languages other than English, primarily Chinese dialects. Medicare or Medicaid and self-pay are the 2 most common forms of payment at this CHC.

### Participants

Study participants include clinic personnel and patients who received RPM devices. Clinic personnel were recruited purposefully based on their knowledge of the RPM implementation effort. Patients eligible for this analysis were adults who received an RPM device as part of the FCC program and used it at least once to upload data to the EHR. Details about how the clinic provided devices to patients are given in the Results; in brief, clinicians had discretion to select patients for the RPM program. The RPM implementation team decided to prioritize distributing RPM devices to individuals with uncontrolled HTN, substantial barriers to care, no evidence of prior smartphone use, and no previous experience with web-based office visits.

### Data Collection

Device distribution data collected by the CHC were used to confirm participants and dates of device disbursement and return, if available. BP measurement data were extracted from the CHC’s EHR. Baseline BP measurements from RPM devices were collected from the time the patients received the devices through day 7. Seven days were added to the initial device distribution date to account for patients’ initial learning curve using the RPM device. Subsequent device use data were collected at months 1, 3, and 6.

Qualitative data included semistructured interviews with 2 clinicians and 2 patients participating in the RPM program at the CHC. See Multimedia Appendices 1 and 2 for interview guides. Interviews were conducted from June 2022 to July 2022 and lasted 30-60 minutes. A clinician champion [22] who led and advocated for the RPM program identified staff and patients to interview based on their RPM involvement. Interviews were held virtually and audio-recorded with permission. Patient interviews were conducted through a professional interpreter. We also gathered background information by conducting informal interviews with OCHIN staff involved with administering and supporting the FCC program and by reviewing documents pertaining to program roll out. Recorded interviews were professionally transcribed, de-identified, and uploaded to NVivo (release 1.7; Lumivero) for analysis.

### Data Analysis

Quantitative data were analyzed descriptively to assess patterns of RPM use in the 6 months following adoption of the tools and patient characteristics associated with these patterns. Paired differences between BP measurements from the RPM devices were assessed using nonparametric Wilcoxon signed-rank tests because of the nonnormality of the data. We assessed how the device’s BP data changed at 0-7, 30-37, 90-97, and 182-189 days post distribution.

Per explanatory sequential design, we assessed what qualitative data explained the quantitatively measured improvement in BP control associated with participation in the RPM program. Qualitative data were analyzed thematically, informed by the CFIR (Consolidated Framework for Implementation Research) [23-25]. First, an initial code list was developed based on key CFIR constructs (eg, process, inner setting, outer setting, and intervention characteristics), with additional codes added inductively. Emerging themes were shared with the OCHIN FCC Program staff, CHC staff interviewees, and the full cross-disciplinary research team to confirm early findings and foster interpretation through diverse perspectives. Results are conveyed using a case study reporting approach characterized by chronological narrative framing.

### Ethical Considerations

The Institutional Review Board at Kaiser Permanente Northwest approved this study (#00000405). All methods were carried out in accordance with relevant guidelines and regulations. BP measurement data were extracted from the OCHIN Epic EHR, as allowable by an agreement with OCHIN member health centers. Informed verbal consent was obtained from interview participants who were notified of their right to refuse to participate and the study team’s procedures for deidentifying data. Patient participants were provided with a $50 gift card in appreciation of their time. The CHC received US $400 as an impact payment in recognition of their participation in the evaluation of their RPM program.

## Results

The CHC in this case study received 50 Bluetooth-enabled BP cuffs and 50 iPhones with unlimited wireless data for 1 year and implementation support as described above. They opted to use a lending model of device distribution to maximize patient reach.

### Device Use and Blood Pressure

BP readings were received from 147 patients, indicating that each of the 50 devices was loaned to an average of 3 patients over the course of the RPM intervention. Patients whose records included device return dates used their devices for an average of 195 days. The CHC provided the study team with a list of patients who received devices (n=152). This list was cross-referenced with EHR data indicating whether the patient used the device. Five patients did not have evidence of device use, leaving 147 patients in the sample. Among the 147 patients, 63% (n=93) were female, 98% (n=144) were Asian, and none were Hispanic. The mean age was 70 years, and 90% of the patients were at or below the federal poverty level.

**Figure 1:**
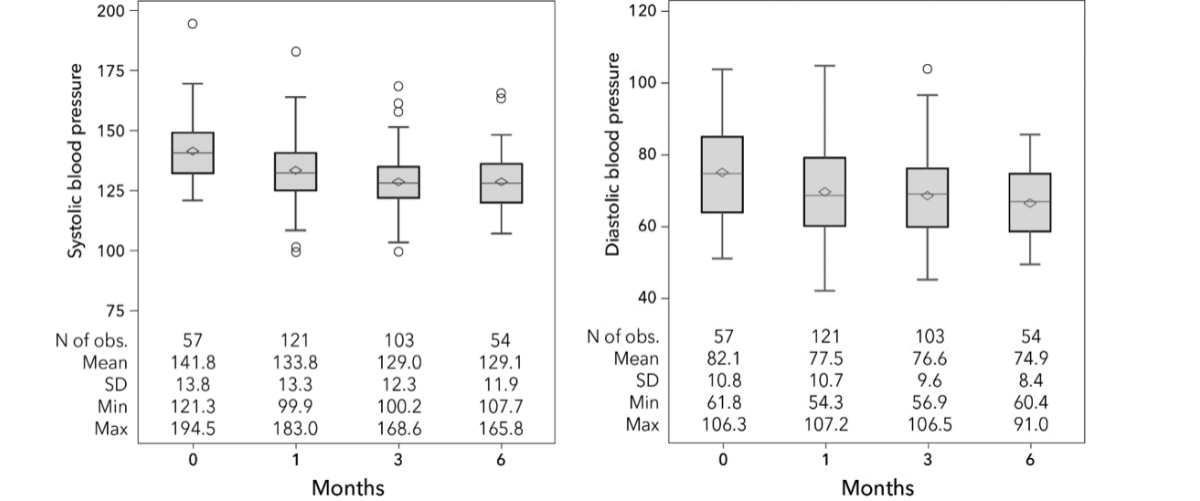
Distribution of mean systolic and diastolic blood pressure by month.

Average BP steadily dropped from baseline to 6-month follow-up, with a reduction of systolic (–13.4 mm Hg) and diastolic (–6.4. mm Hg) measurements among those having BP measurements at 6-month follow-up (Figure 1).

This translated into more than doubling of HTN control (BP<140/90 mm Hg). At baseline, 33.3% of patients with BPs at baseline had controlled HTN, and 81.5% of patients with BPs at 6-month follow-up had controlled HTN.

### RPM Program Implementation

Insights from qualitative data about RPM program implementation illuminated how the clinic successfully implemented RPM. When OCHIN received the FCC grant, it broadly communicated the RPM opportunity across its membership and invited health centers to submit proposals to participate in the RPM program. Proposals were scored based on several domains, including prioritizing health centers and patients most disproportionately affected by COVID-19.

Leaders at the case study CHC convened a team to oversee implementation of the RPM program. This ”RPM team“ consisted of 4 clinicians with an interest in telehealth who agreed to pilot the program, and members of the CHC’s health coaching, quality improvement (QI), and health information technology teams. The team met weekly for 3 months to plan how to implement the RPM program. The QI director developed meeting agendas, tracked task status, and enforced timelines.

The RPM team viewed the program as a ”launch point“ for more broadly shifting their approach to care delivery from episode- or encounter based to a continuous model. Their envisioned new model involved focusing on how patients were doing between visits and enabling care teams to support chronic condition management in a manner convenient for patients. The team saw the value of RPM as part of this vision but knew it required significant planning. Implementation was complicated by the concurrent rollout of a patient EHR portal that did not include the languages predominantly spoken by this CHC’s patients. The RPM team considered which functions of the portal could be useful to RPM patients who were unable to read the available languages given that portal activation was required to send BP data to the care team. The team decided to prioritize distributing RPM devices to patients with multiple challenges to HTN control, as described previously. A clinician reflected,

I was very skeptical because I was like, “It is going to be so hard to do it with our patients.” And at the same time, though, there's this kind of like, “Well, if we're not going to try it, then who will try it?” Just because it's hard, it doesn't mean that our patients can't benefit from it.

MD1

Given the unique needs of the population they were trying to reach, the CHC did not use the implementation support materials provided by OCHIN. Instead, they developed printed materials to support device adoption and use in different languages and literacy levels, and culturally tailored HTN educational materials. One of the clinicians piloting the program shared,

There's a language barrier for our patients, and so we had to figure out what is the most simple document that we can give people that's very pictorial, has a lot of arrows, and not a lot of text. We just came up with a lot of the materials in-house. I think our team was really used to coming up with teaching materials for staff, and so they helped us to make it even more simple.

MD1

The team also developed device distribution and return workflows (ie, handing out and receiving devices back), and an EHR-based dashboard that tracked recruitment, interactions about device troubleshooting, and coaching calls, as well as reports on incoming device data. The RPM team was concerned that patients would not have enough IT support and would experience substantial language and technology literacy barriers, so they trained the clinic’s health coaches, who are also medical assistants, to support patients as required. The CHC’s health IT team provided backup support.

Members of the RPM team with QI roles developed systems for tracking program metrics, which alerted CHC leaders to their initial investment in the program and to the resources likely needed to sustain it. This was primarily done using structured EHR data elements, including when the devices were distributed and returned, how many coaching calls each patient received, and how much troubleshooting each patient required.

Clinicians involved in participating in the RPM program initially reached out to eligible patients to offer the devices; if interest was expressed, a health coach scheduled an hour-long appointment to set up and sync the devices and provide patient education on device use. Health coaches supported patients with portal sign-up, then synced the BP cuff to the provided iPhone using the iHealth app, which interfaced with the Apple Health app that interfaced with the patient portal. BP data automatically went through the patient portal directly to the EHR’s BP data, which could be reviewed by care team members. A member of the IT team developed reports showing average weekly and monthly BP over time. Clinicians and health coaches met regularly to review the BP readings sent from the RPM devices to the EHR, and made plans for patient follow-up, which included potential medication adjustments and web-based appointments with a health coach. Patients were eligible for health coaching if they demonstrated ability to use the devices by having at least 5 BP readings submitted from the devices.

The CHC experienced notable barriers to implementing the RPM program, particularly given the many competing demands related to clinics’ COVID-19 response. Care teams faced challenges in setting up and syncing devices; patients reported difficulty in maintaining synced connections and staying logged in. After some testing and refinement, CHC leaders were pleased with the promising results of the RPM program, a sentiment echoed by the patients. One clinician shared,

We had pretty fantastic results in terms of people's blood pressure becoming under control. We also were able to pick up on things that we never thought we would. I was alerted because [one of my patients] had a very low heart rate, and I couldn't figure out why, so they ended up calling him. It turned out he was prescribed a different medication by another physician that was a beta-blocker that lowers your heart rate. Another one of my patients had been fairly well controlled for a while and then all of a sudden had some spikes in his blood pressure. Our health coach called him, and it turned out he was having a gout attack. And so, our nurse said, ‘You should come in and be treated.’ So he got treated. But we found out through blood pressure. So, I think it definitely works well.

MD2

As care teams noticed improvements in patients’ BP, the CHC added 2 RPM team members to support the program: a digital health advocate to work with patients on device setup and technical support and a program manager to support project management of the RPM activities. Based on the organization’s prioritization of RPM, their grants department wrote a grant proposal to fund the digital health advocate position. They received multiple grants to support the digital health advocate role but also acknowledged the lack of sustainability of grant-funded positions.

Patients said they felt prepared to use the devices after the initial visit with the health coach and were able to call the coach for support with their devices. One patient shared her and her husband’s experience using the devices,

When we first received the devices, the nurse taught me how to use the device in person. And they know that this technology is a little bit difficult for us, so they made sure that we learned the steps and how to use it. And then after a couple of days, my husband forgot how to use it. And then I called the clinic and asked the nurse. And the nurse taught us on the phone again until we all felt comfortable using the devices.

Patient 1

Health center clinicians noted that the dropout rate for the program was high, as approximately 30% of patients who initially said they wanted a device chose not to participate after the initial related visits. Despite implementation and technology barriers, however, the CHC was satisfied with their decision to prioritize RPM and is expanding the RPM program to more care teams in their health system and considering how to sustain the program over time**.**

## Discussion

### Principal Findings

Among adult patients selected to receive a home BP cuff and smartphone, BP reductions were demonstrated during device use, likely due to a combination of factors including increased staff attention to lowering BP, educating patients how to properly use the devices, and motivating patients to actively participate in RPM and HTN management. In addition to measuring BP, motivated patients may have adhered better to lifestyle recommendations and medication prescriptions. Neither the relative contributions of these and other factors, nor long-term durability of HTN control can be accurately assessed from these data, but these would be helpful to analyze in future investigations.

Limited prior research has assessed factors affecting RPM implementation and use in CHCs [26]. These results indicate that successful program implementation in CHCs may involve not just technology and training but also substantial upfront planning and commitment, driven by a clinical champion or leader. This may include establishing an RPM team, developing related workflows, and creating data reports to monitor clinical care decisions, in collaboration with patients. This may be challenging for health centers that lack a robust staffing model with QI, EHR support, health coaching, and other resources available to support pilot testing and material development. For such clinics, a more modest pilot phase might be more feasible, but may not yield the same clinical outcomes.

Prior research showed the potential for technology innovations such as RPM to exacerbate health disparities [27-29], exemplified by inequitable expansion of RPM technologies during the COVID-19 pandemic [30,31]. It is, therefore, important to document successes like the CHC described here, which saw positive outcomes from its RPM program despite its focus on patients facing considerable barriers [32,33]. Elements of its success included developing multilingual materials and using uncomplicated RPM devices to support patient accessibility. Maintaining regular outreach with participating patients (ie, cointervention) also likely helped, as has been demonstrated by others [13,34-36]. Future research is needed to examine the impact of different implementation strategies on the uptake and sustained use of RPM devices, particularly among patients facing barriers to care.

### Limitations

A single case study limits the generalizability of findings; the case study approach was used here to provide a comprehensive view of this multifaceted topic [37]. Also, retrospective studies are limited in that they provide a snapshot in time after an intervention has been implemented but do not provide a detailed understanding of implementation in real time. This potential was mitigated here by collecting data as close to the project end date as possible to optimize the timeliness of reflections about implementation. Finally, because this primarily is a qualitative evaluation, quantitative analysis was limited to descriptive statistics. We compared average BP values of patients with BP data at the various timepoints rather than tracking individual patients. This likely biased our results by excluding those patients who were less actively participating. And since there was no usual care control group with which to compare effectiveness of RPM on BP reduction, more robust statistical analysis could not be performed.

### Conclusions

The experience of one CHC indicates that while RPM technology holds promise for addressing chronic disease management among safety net patients, successful and equitable RPM implementation will require substantial investment in implementation support and technical assistance. Further exploration of these issues in a prospective evaluation is currently underway.

## Appendix

Multimedia Appendix 1 Clinic Staff Interview Guide.

Multimedia Appendix 2 Patient Interview Guide.

## Abbreviations

BP: blood pressure
CARES: Coronavirus Aid Relief and Economic Security
CFIR: Consolidated Framework for Implementation Research
CHC: community health center
EHR: electronic health record
FCC: Federal Communications Commission
HTN: hypertension
QI: quality improvement
RPM: remote patient monitoring

## Data Availability

The data sets used and analyzed during this study are available from the corresponding author on reasonable request.
