# Supplementary material for: Evaluation of a Remote Patient Monitoring Program During the COVID-19 Pandemic: Retrospective Case Study With a Mixed Methods Explanatory Sequential Design
Source: JMIR Form Res. 2024 Jul 9;8:e55732. doi: 10.2196/55732 (PMC11267095; doi:10.2196/55732)
Supplement: Multimedia Appendix 1 [file formative_v8i1e55732_app1.docx]

**REDUCE Clinic Staff Interview Guide**

***INTERVIEWER NOTES***

- *Interviews are meant to be semi-structured.*
- *The questions below represent topics for discussion and are not intended to cover every subject that may come up during the interview; it is also possible that not all listed questions will be asked.*

GENERAL QUESTIONS

1. **Who was involved in the pilot project at your site?**

Probes:

- Who made the decision to participate in the pilot?
- Which staff roles were involved in distributing and monitoring device use?

1. **How did you learn about the FCC remote patient monitoring program?**
2. **What were your initial reactions when you learned about it?**

Probes:

- What were you hoping they would help with?
- How did they address your clinical needs / priorities?

1. **What initial training and orientation did you receive?**
2. **How were patients selected to participate in the pilot project (e.g., HTN status, age, geographic location)?**

Probe:

- Why did you select patients based on that?

1. **Did you feel prepared to use the tools or answer questions about them when they were provided to patients?**

Probes:

- - What do you wish you had known when first using the tools?
  - What types of technical issues did you encounter?
  - How often did patients reach out with questions?
  - What kinds of questions did you get?

1. **How did the tools help achieve chronic disease management? Where did they fall short?**

Probes:

- To what extent were you able to view a patient’s reported measurement?
- How did you use RPM data to inform your clinical decision making?
- Do you have any stories to share about patients who improved their disease management with RPM tools?
- Do you have any stories to share about patients for whom the RPM tools weren’t successful?
- Did your clinic receive as much RPM data as you expected? What do you think impacted that?

1. **What resources or support did you receive during the pilot?**

Probes:

- What could have been improved?
- What went well?

1. **What improvements could make the next round of using the FCC tools more successful?**
