# Supplementary material for: Evaluation of a Remote Patient Monitoring Program During the COVID-19 Pandemic: Retrospective Case Study With a Mixed Methods Explanatory Sequential Design
Source: JMIR Form Res. 2024 Jul 9;8:e55732. doi: 10.2196/55732 (PMC11267095; doi:10.2196/55732)
Supplement: Multimedia Appendix 2 [file formative_v8i1e55732_app2.docx]

**REDUCE Patient Interview Guide**

***INTERVIEWER NOTES***

- *Interviews are meant to be semi-structured.*
- *The questions below represent topics for discussion and are not intended to cover every subject that may come up during the interview; it is also possible that not all listed questions will be asked.*

GENERAL QUESTIONS

Last year, your clinic gave you a blood pressure cuff and iPhone to help monitor your health. These devices were paid for by a grant from the Federal Communications Commission or the FCC. The reason the FCC provided funding for these devices was to help patients continue to receive health care during the Covid-19 pandemic.

1. **Please tell me about your experience receiving and using the devices.**
2. **How did you learn about the devices?**
3. **What motivated you to try them out?**
4. **Initially, did you feel that the devices would be helpful for managing your health condition? Please describe why or why not.**
5. **What instructions or training did you receive about using them?**
6. **How prepared did you feel in getting started with the devices?**
7. **How often did you use the devices?**
8. **Tell me your experience using the iPhone and what it was like to pair your blood pressure monitor with your iPhone app.**
9. **What was easy about using the devices?**

Probes:

- Blood pressure cuff fit around arm, size and location of buttons, screen readability, information storage and retrieval, integration with smartphone app, ability to communicate readings to provider, technology support

1. **What was hard about using the devices?**

Probes:

- Blood pressure cuff fit around arm, size and location of buttons, screen readability, information storage and retrieval, integration with smartphone app, ability to communicate readings to provider, technology support

1. **What were you hoping that the devices would help with?**

Probes:

- How did that turn out?
- Any surprises or disappointments?

1. **Did you reach out to get help on using the devices?**

Probe:

- Who helped you with this?

1. **What support could have helped in using the devices more than you did?**
2. **What other suggestions do you have for improving how these devices are used in the future?**
